# Experience, cortisol reactivity, and the coordination of emotional responses to skydiving

**DOI:** 10.3389/fnhum.2015.00138

**Published:** 2015-03-25

**Authors:** Vanessa J. Meyer, Yoojin Lee, Christian Böttger, Uwe Leonbacher, Amber L. Allison, Elizabeth A. Shirtcliff

**Affiliations:** ^1^Department of Human Development and Family Studies, Iowa State UniversityAmes, IA, USA; ^2^Department of Psychiatry, Tulane UniversityNew Orleans, LA, USA; ^3^Department of Social Psychology, University of InnsbruckInnsbruck, Austria; ^4^Department of Psychology, University of New OrleansNew Orleans, LA, USA

**Keywords:** cortisol, habituation, skydiving, anxiety, emotion, HPA axis

## Abstract

Physiological habituation to laboratory stressors has previously been demonstrated, although the literature remains equivocal. Previous studies have found skydiving to be a salient naturalistic stressor that elicits a robust subjective and physiological stress response. However, it is uncertain whether (or how) stress reactivity habituates to this stressor given that skydiving remains a risky, life-threatening challenge with every jump despite experience. While multiple components of the stress response have been documented, it is unclear whether an individual’s subjective emotions are related to their physiological responses. Documenting coordinated responsivity would lend insight into shared underlying mechanisms for the nature of habituation of both subjective (emotion) and objective (cortisol) stress responses. Therefore, we examined subjective emotion and cortisol responses in first-time compared to experienced skydivers in a predominantly male sample (total *n* = 44; males = 32, females = 12). Hierarchical linear modeling (HLM) revealed that experienced skydivers showed less reactivity and faster recovery compared to first-time skydivers. Subjective emotions were coordinated with physiological responses primarily within first-time skydivers. Pre-jump anxiety predicted cortisol reactivity within first-time, but not experienced, skydivers. Higher post-jump happiness predicted faster cortisol recovery after jumping although this effect overlapped somewhat with the effect of experience. Results suggest that experience may modulate the coordination of emotional response with cortisol reactivity to skydiving. Prior experience does not appear to extinguish the stress response but rather alters the individual’s engagement of the HPA axis.

## Introduction

Although dysregulation of the hypothalamic-pituitary-adrenal (HPA) axis has been associated with both mood and anxiety disorders, our understanding of the relationship between acute endogenous stress hormone responsivity and subjective measures of mood and anxiety is primarily driven by experiments with laboratory stressors (Jezova et al., 2004; Kudielka et al., 2004; Young et al., 2004). Jumping out of an airplane, similarly to laboratory-based stressors, reliably elicits a subjective and physiological stress response, including activation of both the autonomic nervous system and HPA axis (Schedlowski et al., 1992; Dugué et al., 1993; Aloe et al., 1994; Benschop et al., 1996; Richter et al., 1996; Chatterton et al., 1997; Deinzer et al., 1997; Biondi and Picardi, 1999; Hynynen et al., 2009; Plenis et al., 2011; Taverniers et al., 2011; Yonelinas et al., 2011; Hare et al., 2013). Additionally, skydiving has been shown to independently cause responsivity of other biomarkers, including increasing levels of DHEA (Oberbeck et al., 1998), epinephrine and norepinephrine (Schedlowski et al., 1993; Benschop et al., 1996; Richter et al., 1996), nerve growth factor (Aloe et al., 1994), and lymphocytes (Schedlowski et al., 1993; Benschop et al., 1996). Skydiving is a unique model to study acute emotional and physiological stress in humans for several reasons. First, skydiving is distinct from laboratory stressors in that it is a more intense challenge than would be possible in a laboratory (Yonelinas et al., 2011). Second, although in-laboratory stressors may be uncomfortable, skydiving carries an actual risk of injury or death, making it a salient and ecologically valid stressor. Third, skydiving is socially-sanctioned and generally perceived as an enjoyable activity (Franken et al., 2006), in contrast to aversive psychosocial or pain-associated stressors.

Examining reactivity to a salient, ecologically valid stressor such as skydiving allows for the investigation of the nature of stress response habituation. Currently, it is still unclear how prior experience modulates stress responses. Physiological habituation to laboratory stressors has been observed but this research is not fully robust (Kirschbaum et al., 1995b; Schommer et al., 2003; Petrowski et al., 2012). Prior research on habituation to skydiving has been arguably even more equivocal. Autonomic reactivity to skydiving shows habituation (Hynynen et al., 2009) although the differences between novice and experienced jumpers may be subtle (Allison et al., 2012). The cortisol response to skydiving has been shown to habituate to successive jumps in one day (Deinzer et al., 1997). Conversely, some studies have not found habituation of cortisol reactivity (Schedlowski et al., 1992; Hare et al., 2013). Subjective anxiety may habituate (Hare et al., 2013), suggesting the relationship of subjective emotion and physiological reactivity may be different with experience.

Previous research has indirectly pointed toward a shared mechanism underlying subjective anxiety and cortisol levels, although directly examining the coordination of subjective response and cortisol reactivity or recovery (or how this relationship may change with experience) remains under-studied. A group of military parachutists dichotomized into high and low anxiety groups demonstrated no difference in post-jump cortisol level indicating subjective anxiety may be unrelated to cortisol response (Schedlowski et al., 1992). Novice and experienced parachutists were combined, thereby preventing examination of how this relationship may change with experience. Trait anxiety measured three days before skydiving was not a significant predictor of post-jump cortisol (Chatterton et al., 1997); therefore personality- level anxiety may not be related to an individual’s cortisol response to a specific event. Subjective anxiety on the day of the jump was not assessed, which precludes comparing temporally-relevant subjective anxiety to cortisol response. Some evidence suggests a discordance between subjective anxiety and cortisol response among experienced skydivers (Thatcher et al., 2003; Hare et al., 2013); however, it is not clear how subjective anxiety may relate to the shape of the cortisol response (e.g., reactivity or recovery), particularly in first-time jumpers. The novel contribution of the current study was to directly examine the coordination of subjective responses to the stressor with cortisol reactivity and recovery. To reconcile prior inconsistencies regarding habituation, we examined how this relationship may differ between first-time and experienced skydivers.

The present study aimed to reconcile previous disparate findings by examining the subjective (emotional) and objective (cortisol) stress responsivity to skydiving in first-time and experienced skydivers. The aim of this investigation was to advance the understanding of how subjective or objective habituation to a salient stressor may occur, and how those responses may be related. We utilized the robust statistical method of hierarchical linear modeling (HLM) which simultaneously modeled the entire cortisol response within a single model to determine if habituation of any component of the cortisol response is apparent (and independent of the other components). We hypothesized that highly experienced jumpers would show an attenuated cortisol response, compared to first-time skydivers, given their extensive previous exposure to the stimulus. Additionally, we hypothesized that higher subjective anxiety immediately before jumping may be associated with a steeper incline in cortisol leading up to the jump, specifically among first-time skydivers. As an exploratory aim, we proposed to examine the relationship of cortisol response to subjective happiness following the jump. Skydiving is known to elicit strong positive emotions (Celsi et al., 1993; Roth et al., 1996; Price and Bundesen, 2005); although how this subjective emotion may relate to physiological activation has not been investigated.

## Methods

### Participants

The final sample of 44 participants (mean age = 29.6; 32 males, 12 females) included 29 first-time jumpers (mean age = 28.3; 18 males, 11 females) and 15 experienced jumpers (mean age = 32.1, 14 males, 1 female). First-time jumpers completed tandem skydives, the conventional and safest method for inexperienced jumpers. Experienced skydivers had a median number of 208 previous jumps (range 23–8000) and completed solo skydives. Skydivers were recruited at Gold Coast Skydivers in Lumberton, MS. Individuals who came to the facility to skydive were invited to participate. Exclusion criteria included obvious/reported health complications or age outside of 18–50 years (average age = 29.59, *SD* = 7.60). Our convenience sampling method lead to the gender imbalance between groups, because during data collection more males than females came to the skydiving facility. The protocol was approved by the University of New Orleans Institutional Review Board.

### Procedures

Participants jumped in the afternoon to diminish the impact of diurnal rhythms on the cortisol response (Dickerson and Kemeny, 2004). Saliva samples were collected from participants at 5 time points. Sample 1 was collected 1.5-h prior to the skydive, shortly after arrival at the facility. Sample 2 was collected 15-min prior to the skydive. At the time of their skydive (average = 2:17 pm, range 12:43 pm–3:46 pm), participants boarded the plane and ascended to an exit altitude of 14,000 feet. All participants (both first-time and experienced) exited from the same plane at the same altitude. Freefall lasted for one minute, followed by a 5 min parachute descent. Samples 3, 4 and 5 were collected immediately after, 15-min and 60-min after landing, respectively. Due to time constraints, experienced jumpers only provided samples 1, 3, and 4. Our ecologically valid design forced us to adapt data collection to a typical day for the experienced jumpers, which includes practicing on the ground, packing parachutes, and performing gear safety checks. Sample times were centered on time of exit from the aircraft.

### Cortisol

Saliva was assayed by Middleton Research using a commercially available enzyme immunoassay from Salimetrics (State College, PA). Intra-assay coefficient of variance (CV) was below 7% and inter-assay CV was below 15%. Cortisol values were log transformed to normalize the data.

### Subjective Emotion

At the time of each saliva collection, visual analog scale subjective emotion ratings were collected by having participants indicate how anxious they felt “right now”, by placing a mark on a 16-cm line between “not at all anxious” and “highly anxious”. A parallel question queried happiness. Visual analog scales are advantageous for minimizing constraints on participant responses and are less restrictive than Likert-type scales. The single-item measure captured overall momentary emotion while minimizing demand on participants. Single-item VAS assessments have demonstrated validity, reliability, and high correlation with more extensive emotion measures (Abdel-Khalek, 2006; Davey et al., 2007). Mean emotion ratings were created for each individual by averaging across their responses from all time points. Emotion ratings were then centered on the mean and these centered values were used for HLM analyses. This approach allows us to capture changes in emotion relative to each individual’s average.

### Statistical Analyses

We examined first whether first-time and experienced jumpers showed a difference in pre-jump anxiety and post-jump happiness ratings, using a one-way analysis of variance (ANOVA) with experience (first-time vs. experienced) as the between-subjects variable and each emotion rating as the within-subjects variable across time. These ANOVAs established which time point of each emotion was most important.

Hierarchical linear modeling (HLM) analyses investigated the cortisol response to skydiving. HLM analyses take into account the nested structure of the hormone assessments, where every person has several measurements (Raudenbush and Bryk, 2002). Not only does this allow examination of the shape of the hormonal response, it preserves the statistical power from having multiple samples within each individual (Hruschka et al., 2005). While our sample size is modest, we have several observations for each individual which increases our statistical power (Raudenbush and Xiao-Feng, 2001). HLM is commonly used with sample sizes of the magnitude of the present study (Adam, 2006; Fries et al., 2008). With HLM we simultaneously model all components of the cortisol response (peak, reactivity, recovery) as an integrated profile, and therefore control for each of those components. This type of analysis generates a highly conservative estimate of peak, reactivity, and recovery. Moreover, HLM is robust to missing data or variable data, which is important in the present study where groups provided different numbers of samples and collection times varied from one individual to another.

In all HLM analyses, cortisol was the predicted variable and our time variables (time to jump, time since jump) were the Level 1 predictors. Our first model had experience (first time vs. experienced) as the Level 2 predictor. Our emotion models used centered emotion ratings (anxiety or happiness) as Level 2 predictors.

Potential confounders were also entered as Level 2 predictors. Both age and sex were entered into the model and evaluated for their impact. To examine the potential effect of medication use, we grouped medications into five categories: (1) non-narcotic anti-inflammatories, (2) antibiotics/non-steroidal cold, allergy, and asthma medications, (3) psychotropics, (4) oral contraceptives; and (5) others (Schreiber et al., 2006). No participants were taking oral or non-oral steroids. Each medication class was entered as a level 2 predictor to evaluate influence on the model.

## Results

### Emotion Ratings

**Figure 1:**
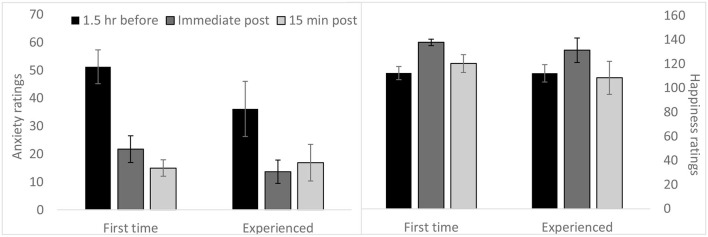
**Subjective emotion ratings among first-time and experienced skydivers**. Anxiety and happiness ratings among first-time and experienced jumpers at three time points across the skydiving day confirms that peak anxiety occurred at 1.5 h before the jump and happiness peaked immediately after the jump. This justifies examining these emotions specifically at those time points. Error bars indicate +/− one standard error. Range of possible values 0–160.

**Figure 2:**
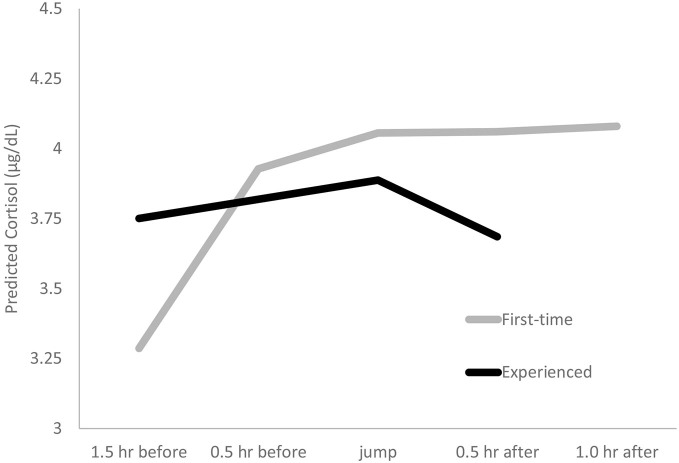
**Cortisol response in first-time and experienced skydivers**. Predicted cortisol is log-transformed cortisol (μg/dL) predicted by our HLM model. Experienced jumpers provided samples at time 1, time 3 (jump), and time 4. First-time jumpers provided samples at all five time points.

We found no difference between first-time and experienced on pre-jump ratings of anxiety, *F*_(1,42)_ = 2.52, *p* = 0.12 (see Figure 1), or on post-jump ratings of happiness, *F*_(1,42)_ = 0.29, *p* = 0.60 (see Figure 2). Across both groups, anxiety levels were highest upon arrival at the facility, *F*_(1,43)_ = 27.47, *p* < 0.0001, then decreased thereafter. Happiness ratings were highest immediately after landing, *F*_(1,43)_ = 28.965, *p* < 0.0001, as expected. The average anxiety rating decreased 50% from before the jump to after the jump and average happiness rating increased 41% from before the jump to immediately after landing. Given that the first time point was the peak in anxiety ratings, and the third time point was the peak in happiness ratings, we selected these time points for our cortisol-emotion analyses. Anxiety at time 1 was centered on the individuals’ mean anxiety ratings and happiness at time 3 was centered on the individuals’ mean happiness ratings.

### Cortisol Response

HLM analyses investigated change in cortisol concentrations in response to skydiving. An initial base model indicated how much of the variance in cortisol was due to inter-individual variability vs. intra-individual variability or moment to moment changes in the stress hormone. We found that 54% of variance was accounted for by differences between individuals and 46% of the variance was accounted for by momentary fluctuations within an individual, ${\text{χ}}_{\left(\text{21}\right)}^{\text{2}}$ = 112.87, *p* < 0.001. Across both groups, cortisol increased before the jump, *β* = 0.44, *t*_(43)_ = 5.94, *p* < 0.001, and peaked soon after the jump, *β* = 2.49, *t*_(43)_ = 16.51, *p* < 0.001. Across all participants, 84% (*n* = 37) showed increased cortisol in response to the skydive including 75% (*n* = 33) whose cortisol increased by more than 15%. The average cortisol response was an increase of 45% above sample 1. While we focused on a continuous measure of reactivity, a method of describing reactivity is by categorizing reactivity status. Of the 16% cortisol non-responders, six out of the seven were experienced jumpers, a significant group difference, ${\text{χ}}_{\left(\text{1}\right)}^{\text{2}}$ = 9.89, *p* > 0.01.

Although there was not a difference in jump time for first-time and experienced jumpers (*p* > 0.05), we accounted for time of day in our model by adding jump time as a Level 2 predictor. We examined experience by including the group variable (first-time vs. experienced) to test for group differences. When accounting for time of day, experienced jumpers showed less reactivity, *β* = −0.42, *t*_(41)_ = −2.61, *p* = 0.01, and faster recovery, *β* = −0.48, *t*_(203)_ = −2.16, *p* = 0.03 than first-time jumpers (see Figure 2). When potential covariates (age, sex, medication use) were entered into the model, they had no impact on cortisol level, reactivity, or recovery (*p*’s > 0.05). To ensure the differential numbers of samples in each group were not contributing to group differences, we ran our HLM model using data from time points 1, 3, 4 only and found the same pattern of effects where experienced jumpers showed less reactivity, *β* = −0.38, *t*_(59)_ = −2.32, *p* = 0.01, and faster recovery, *β* = −1.44, *t*_(42)_ = −4.64, *p* < 0.001, than first-time jumpers.

We were interested in how pre-jump anxiety was related to cortisol reactivity. Pre-jump anxiety significantly predicted cortisol reactivity in our full sample, *β* = 0 0.005, *t*_(41)_ = 2.17, *p* = 0.04. This effect was driven by the first-time jumpers, where higher pre-jump anxiety predicted increased cortisol reactivity, *β* = 0.009, *t*_(171)_ = 2.64, *p* < 0.001. This pattern was not observed within experienced jumpers, *β* = −0.005, *t*_(13)_ = −0.38, *p* = 0.71.

Next, we entered the post-jump happiness rating as a predictor of cortisol recovery in the full sample. Higher post-jump happiness predicted faster cortisol recovery, at trend level only, *β* = −0.006, *t*_(160)_ = −1.88, *p* = *0*.062. When we did not control for experience (removed first-time/experienced variable from the predictors), post-jump happiness became a significant predictor of faster cortisol recovery, *β* = −0.008, *t*_(161)_ = −2.15, *p* = 0.03. This effect of happiness on faster cortisol recovery held when controlling for anxiety, *β* = −0.008, *t*_(160)_ = −2.19, *p* = 0.03.

## Discussion

This study directly examined the relationship of cortisol reactivity and recovery to subjective responses to a naturalistic stressor. To reconcile prior inconsistencies regarding habituation, we examined how this relationship may differ between first-time and experienced skydivers. We found that first-time skydivers demonstrated increased cortisol reactivity leading up to the jump and slower recovery after landing than experienced jumpers. We found that among first-time jumpers, higher levels of subjective anxiety before the jump predicted increased cortisol reactivity. This coordination of emotional and physiological response was not seen in experienced skydivers. A trend for participants who reported greater subjective happiness after the jump to show faster cortisol recovery was also apparent. Our findings contribute to the habituation literature by demonstrating that prior experience may alter the activation of the HPA axis and change how emotional response is coordinated with physiological reactivity.

### Cortisol Response

Prior literature on the potential for HPA habituation to skydiving has been mixed (Deinzer et al., 1997; Schommer et al., 2003; Petrowski et al., 2012; Hare et al., 2013). The current study utilized a statistical method, HLM, to investigate the shape of the hormonal response, specifically cortisol rise leading up to the jump (reactivity), cortisol level at jump time (peak), and the decrease after the jump (recovery). Although prior studies collected several cortisol assessments on each individual, they but did not take full advantage of this study design as we do in the current study by utilizing HLM to examine the shape of the hormonal response. Within the present study, experienced skydivers demonstrated flatter cortisol reactivity; however, even after hundreds of jumps they still showed increased subjective anxiety and elevated cortisol leading up to the jump. The finding that experienced jumpers cortisol response profile was flattened compared to first-time jumpers is similar to studies that found evidence of habituation (Deinzer et al., 1997). Intriguingly, even after hundreds of prior exposures, the experienced jumpers still showed a significant cortisol response to the jump, similar to findings that show HPA reactivity (Schedlowski et al., 1992; Hare et al., 2013). This helps rectify prior mixed studies by illustrating that habituation to a naturalistic stressor is complex. Experience did not extinguish the cortisol response to skydiving, but rather altered engagement of the HPA axis. This calls into question whether subjective emotional response may help to explain this nuanced HPA habituation.

### Emotional Response

We found dynamic changes in emotion ratings on the skydiving day, with peak anxiety upon arrival to the facility and peak happiness immediately after landing. Prior experience did not impact emotional responses to jumping. This contrasts with a previous study that found anxiety before skydiving was negatively related to experience, such that more experienced jumpers had lower subjective anxiety compared to first-time jumpers (Price and Bundesen, 2005); however, it is possible that the larger sample size (*n* = 105), allowed for the detection of subtle differences in emotion. Intriguingly, we found the relationship of emotion ratings to HPA activity was moderated by prior skydiving experience. We revealed that HPA functioning was more coordinated with emotion for first-time jumpers than experienced jumpers. This finding fits with previous research demonstrating a discordance between self-reported anxiety and physiological stress reactivity in experienced skydivers (Hare et al., 2013). We speculate that experience may allow individuals to dissociate subjective anxiety and HPA axis activation through improved emotion regulation and cognitive reappraisal (Carlson et al., 2012).

We speculate that extensive prior experience with a stressor may shift the activation of emotion-regulation neurocircuitry. The amygdala, part of the limbic system in the brain, plays a critical role in processing fear responses and threat-related information (Öhman, 2005). The amygdala is also involved in activation of the HPA axis in response to stress and is an important regulator of glucocorticoid secretion (Herman and Cullinan, 1997). In humans, having greater psychosocial resources is related to lower cortisol response, less amygdala, and greater prefrontal cortical activation in response to stress (Taylor et al., 2008). An extensive neural network including the amygdala and prefrontal cortex is involved in emotion regulation (for a review see Ochsner et al., 2012). Having prior experience to draw upon may allow the experienced skydivers to exert greater cognitive control over emotion, reducing cortisol reactivity and dissociating subjective and objective responses to the stressor.

Finally, we found some evidence that subjective happiness after the jump was related to the cortisol response. Given that skydiving can be contextualized as a pleasurable activity (for individuals who chose to skydive as we studied) (Franken et al., 2006), this intense stressor provided a unique opportunity to explore how HPA response may relate to subjective reward. An expanding area of research investigates the overlap of the HPA axis with reward neural circuitry and reward-seeking behavior (Koob and Kreek, 2007). High cortisol has been associated with enhanced reward sensitivity (Oswald et al., 2005) and dopamine is released in the ventral striatum proportionally to cortisol reactivity to a stressor (Pruessner et al., 2004). Conversely, reward has been associated with reduced cortisol reactivity (Creswell et al., 2013). The systems appear to be intricately related, and skydiving proffers a unique opportunity to examine how cortisol response relates to subjective reward. Skydiving elicits a nonpharmalogically-induced reward or “natural high” and shows similarities with addictive behaviors (Celsi et al., 1993; Price and Bundesen, 2005; Franken et al., 2006). Since skydiving elicits both activation of the HPA axis as well as a “natural high”, it is a logical model to investigate the interaction of physiological response with subjective reward. We found that individuals who reported a strong positive emotional response to skydiving demonstrated a faster decrease in cortisol after the jump, lending support for the intricate relationship between stress and reward systems.

Our study has limitations. Although this study has the largest sample size to date (*n* = 44), our group sizes were still modest. Additionally, the number of first-time jumpers was nearly twice as high as the number of experienced jumpers (29 vs. 15). This may have decreased our ability to find a relationship between subjective anxiety and cortisol response in our experienced jumpers. Second, only three time points were collected on the experienced jumpers, compared to the five time points collected on our first-time jumpers, giving us a less complete picture of their cortisol response. However, all physiologically critical time points were captured on all groups (baseline, reactivity, and recovery) and when the HLM models were run examining only the three time points captured on all participants, the pattern of effects was the same. Third, while we had identical jump types between groups (all participants jumped from 14,000 feet from the same airplane, with one minute of freefall before deploying the parachute), first-time jumps were tandem jumps but the experienced group jumped solo. It is possible that the tandem instructors could have provided social support to the first-time jumpers. However, while social support provided by a best friend or romantic partner attenuates cortisol reactivity to a psychosocial stressor (Kirschbaum et al., 1995a; Heinrichs et al., 2003), social support given by a stranger does not (Kirschbaum et al., 1995a). Given that the first-time jumpers had never before met their tandem instructors, it is unlikely any social support provided actually attenuated cortisol response to the jump. Additionally, differing jump types is inherent in this naturalistic model of stress; prior studies comparing novice to experienced skydivers also had differences in jump types. In prior studies, novices jumped from a lower altitude with no freefall and automatically deploying parachutes while experienced jumpers exited at higher altitudes and experienced freefall before deploying their parachutes (Schedlowski et al., 1992; Hare et al., 2013). These are the typical conditions for the contexts in which the data was collected, specifically military parachuting (Schedlowski et al., 1992) and skydiving in the United Kingdom (Hare et al., 2013). As in the current study, these conditions are reflective of how these individuals would be jumping outside of a research study, thereby preserving ecological validity. However, we acknowledge this potential confounder as a limitation and cannot rule out that different jump types could be contributing to the group differences we observed. An interesting future direction for this line of research is to compare responsivity in first-time solo full-altitude jumpers with experienced jumpers.

In conclusion, this study is the first to directly model the relationship of subjective emotional response and cortisol reactivity to skydiving, and how this relationship changes with prior experience. In the context of a naturalistic, salient stressor we found that higher levels of self-reported anxiety were related to greater cortisol reactivity among first-time, but not experienced, jumpers. Our findings suggest that experience alters emotional and physiological arousal but does not extinguish reactivity to an extreme challenge such as skydiving. These changes in psychological and biological responses to a real-life stressor after repeated exposures highlights the adaptability of the human stress response system and points to a dissociation of emotion and physiology with chronic exposure.

## Conflict of Interest Statement

The authors declare that the research was conducted in the absence of any commercial or financial relationships that could be construed as a potential conflict of interest.
